# Impacts of an Electronic Health Record Transition on Veterans Health Administration Health Professions Trainee Experience

**DOI:** 10.1007/s11606-023-08283-4

**Published:** 2023-10-05

**Authors:** Ellen A. Ahlness, Brianne K. Molloy-Paolillo, Julian Brunner, Sarah L. Cutrona, Bo Kim, Erin Matteau, Seppo T. Rinne, Edward Walton, Edwin Wong, George Sayre

**Affiliations:** 1 Center of Innovation for Veteran-Centered and Value-Driven Care, Seattle. VA Medical Center, Seattle, WA USA; 2 Center for Healthcare Organization and Implementation Research, Bedford VA Medical Center, Bedford, MA USA; 3 Center for the Study of Healthcare Innovation, Implementation & Policy, VA Greater Los Angeles Health Care, Los Angeles, CA USA; 4 Division of Health Informatics & Implementation Science, Department of Population and Quantitative Health Sciences, University of Massachusetts Chan Medical School, Worcester, MA USA; 5 Center for Healthcare Organization and Implementation Research, VA Boston Health Care System, Boston, MA USA; 6 Department of Psychiatry, Harvard Medical School, Boston, MA USA; 7 VA Office of Academic Affiliations, Washington, DC USA; 8 The Pulmonary Center, Department of Medicine, Boston University, Boston, MA USA; 9 University of Washington School of Public Health, Seattle, WA USA

**Keywords:** EHR transitions, HPT, mixed methods, residents, students, supervisors, user experience, VHA.

## Abstract

**Background:**

Adoption of electronic health care records (EHRs) has proliferated since 2000. While EHR transitions are widely understood to be disruptive, little attention has been paid to their effect on health professions trainees’ (HPTs) ability to learn and conduct work. Veterans Health Administration’s (VA) massive transition from its homegrown EHR (CPRS/Vista) to the commercial Oracle Cerner presents an unparalleled-in-scope opportunity to gain insight on trainee work functions and their ability to obtain requisite experience during transitions.

**Objective:**

To identify how an organizational EHR transition affected HPT work and learning at the third VA go-live site.

**Design:**

A formative mixed-method evaluation of HPT experiences with VHA’s EHR transition including interviews with HPTs and supervisors at Chalmers P. Wylie VA Outpatient Clinic in Columbus, OH, before (~60 min), during (15–30 min), and after (~60 min) go-live (December 2021–July 2022). We also conducted pre- (March 2022–April 2022) and post-go live (May 2022–June 2022) HPT and employee surveys.

**Participants:**

We conducted 24 interviews with HPTs (*n*=4), site leaders (*n*=2), and academic affiliates (*n*=2) using snowball sampling. We recruited HPTs in pre- (*n*=13) and post-go-live (*n*=10) surveys and employees in pre- (*n*=408) and post-go-live (*n*=458) surveys.

**Approach:**

We conducted interviews using a semi-structured guide and grounded prompts. We coded interviews and survey free text data using a priori and emergent codes, subsequently conducting thematic analysis. We conducted descriptive statistical analysis of survey responses and merged interview and survey data streams.

**Key Results:**

Our preliminary findings indicate that the EHR transition comprehensively affected HPT experiences, disrupting processes from onboarding and training to clinical care contributions and training-to-career retention.

**Conclusions:**

Understanding HPTs’ challenges during EHR transitions is critical to effective training. Mitigating the identified barriers to HPT training and providing patient care may lessen their dissatisfaction and ensure quality patient care during EHR transitions.

**Supplementary Information:**

The online version contains supplementary material available at 10.1007/s11606-023-08283-4.

## Introduction

Although electronic health care record (EHR) transitions are highly disruptive to organizations,^1–3^ few studies have considered their impact on health professions trainees’ (HPTs) experiences (e.g., adversely affecting learning and supervision ^4–6^) despite their critical clinical role within health systems. Trainees (students, interns, residents) have unique needs requiring targeted examination (e.g., short episodic rotations that may occur across EHR transition stages, varied exposure to change management efforts, rotations in underserved settings). To minimize disruptions and ensure HPTs’ ability to contribute to safe, high-quality patient care, additional research is needed to explore HPT experiences and best practices for supporting trainees during organizational EHR transitions. Guided by our research question—*what are the experiences of HPTs during organizational EHR transitions?—*our evaluation examines how a large-scale EHR transition impacts HPT work and learning. EHR transition literature has primarily explored impacts on employees (new work^7^, unfavorable workflows^8,9^, burnout^10^), yet HPTs’ characteristics make them particularly vulnerable to workflow disruptions.

The Veterans Health Administration’s (VA) ongoing transition from its homegrown EHR (CPRS/VistA) to the commercial Cerner Millennium (Cerner) is the world’s largest organizational EHR transition. This process, anticipated to take 10+ years and cost $39+ billion^11^, presents an unparalleled opportunity to understand EHR transition repercussions on HPT experiences due to its scope. VA comprises the nation’s largest program for physicians and 60 other clinical disciplines, educating ~113,000 trainees annually at 150+ facilities with 1400+ academic affiliates.^12,13^ HPTs play a key role in providing patients’ clinical needs.^14^ This transition, affecting numerous trainees, is a rare opportunity for insight. Here, we explore how the VA’s EHR transition has affected HPTs’ ability to learn and work at the third VA transition site.

## Methods

### Study Design

This work is part of SCHOLAR, an ongoing mixed methods formative evaluation of HPT EHR transition experience at VA. This paper focuses on data collected around transition day (“go-live”) at the third VA EHR transition site, Chalmers P. Wylie VA Outpatient Clinic in Columbus, OH (“Columbus”). Columbus is a small ambulatory medical center employing ~1500 clinicians and staff, serving ~44,000 veterans a year through outpatient services, including primary care, mental health, eye care services, pain management, and women’s health.^15^ ~200 HPTs from 27 programs rotate through Columbus annually; we identified eligible HPTs (*n*=57, those training during go-live) from a VA Office of Academic Affiliations (OAA) database. Interview and survey samples had some participant overlap.

### Qualitative

#### Recruitment

We conducted 24 interviews across HPTs, site leaders with supervisory responsibilities, and individuals representing HPTs’ VA Academic Affiliate institutions (henceforth “academic affiliates”) (Table 1). Initial participants were identified by site service leads and recruited via email; snowball sampling identified additional participants. Interviewees not paid by VA received gift cards as thanks.

**Table 1:** Columbus Qualitative Data Collection

	1-month pre-go-live interviews	1-2 week post-go-live interviews	2-month post-go-live interviews	Total
Site leaders*	2	3	1	6
HPTs**	4	4	3	11
Academic affiliates	2	2	3	7
Total	8	9	7	24

*MDs, Pharm.Ds, PAs, APRNs

**Residents, students, interns. Given small Columbus/specific clinical practice HPT sample sizes, clinical area withheld for participant anonymity

#### Data Collection

**Figure 1:**
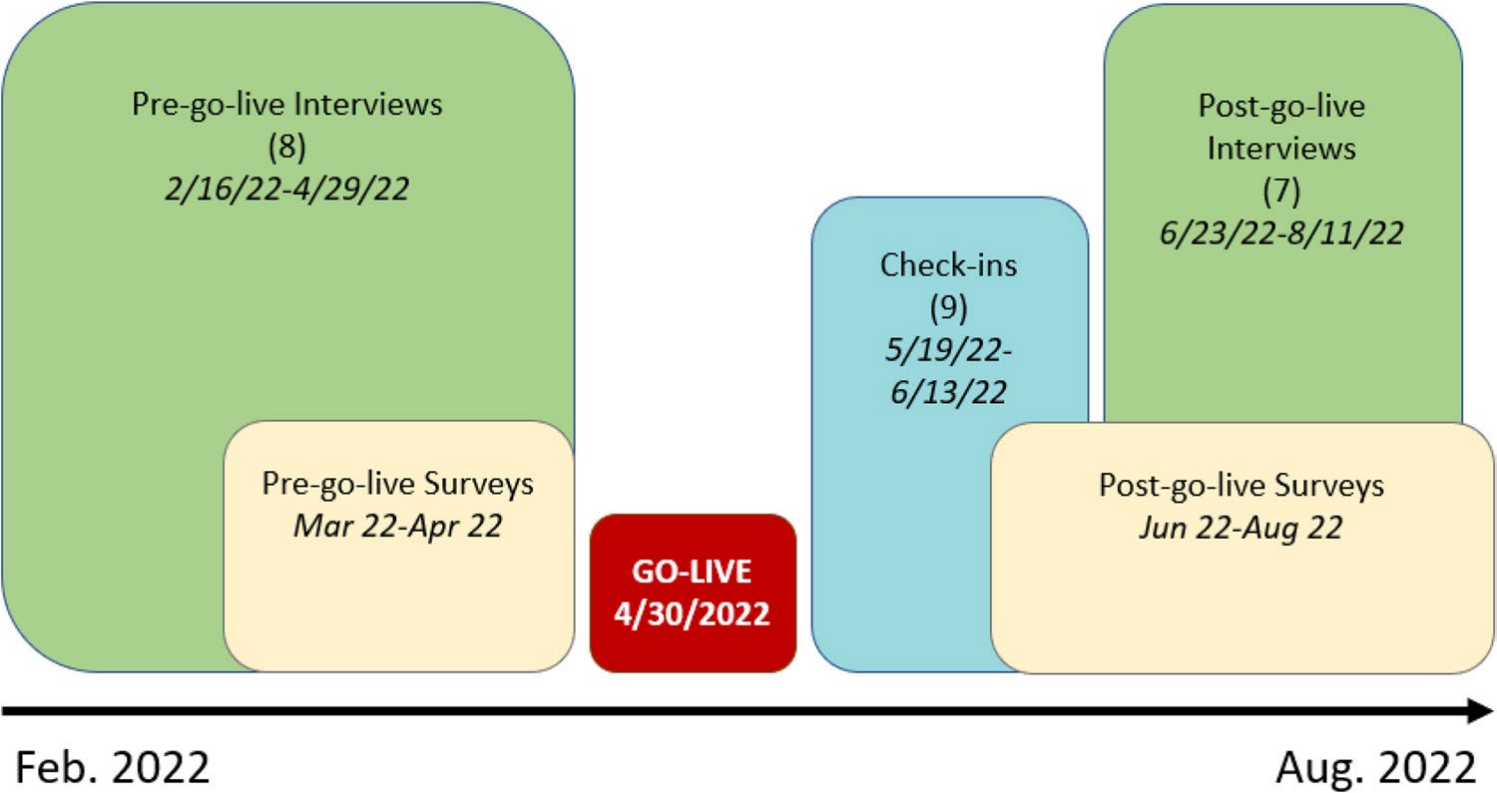
Data collection timeline for pre/post-go-live interviews and surveys from February to August 2022. The green boxes indicate timeframes for qualitative interviews. The teal box indicates the timeframe for qualitative check-ins. The yellow boxes indicate timeframes for survey data collection. The red box indicates the go-live date for the VA facility.

Interview guides were based upon emergent themes from scoping interviews with VA HPT supervisors and site leaders from the first go-live site (Spokane, WA). Interviews were conducted across-go-live (Fig. 1) to capture breadth of end-user experiences across the transition. Qualitative researchers conducted semi-structured interviews with participants pre- (~60 min), during (15–30 min), and post-go-live (~60 min) via Microsoft Teams. Interviewees were informed on study design, participant rights, and provided verbal consent. Interviews integrated grounded probes^16^ to elicit detail (Appendix 1).

#### Data Analysis

Interviewers completed post-interview debrief notes, summarizing content and thematic fit for subsequent team analysis. Data analyses were conducted using deductive and inductive content analysis methods.^17^ Interview transcripts and survey free text responses were coded in ATLAS.ti using a combination of a priori code categories (based on study aims and literature) and emergent codes (based on inductive content analysis: open coding to capture data outside a priori categories allowed identification of new themes). Data was aggregated across time points to identify salient experiences. Qualitative researchers met weekly to discuss data and reach consensus on theme/finding interpretation; given the sample size, all data was coded and analyzed in lieu of stopping at a point of saturation. Simultaneously collected qualitative and quantitative data were compared to reach findings through mixed-method data merging.^18^

### Quantitative

#### Survey Development

We conducted pre- and post-go-live surveys to ascertain HPTs’ experiences preparing for and using the new EHR, and the transition’s impact on their overall clinical training experiences. Survey questions were informed by emergent themes from pilot interviews with VA HPT supervisors and site leaders from the first go-live site.

#### Data Collection

SCHOLAR surveys queried HPTs’ clinical and educational training experiences in relation to the EHR transition (Table 2). All 57 Columbus HPTs were emailed invitations, SCHOLAR surveys, and three follow-up reminders. The voluntary, anonymous surveys took ~10 min to complete. HPTs completing the survey (pre-go-live *n*=13; post-go-live *n*=10) could enter a lottery to win a $100 gift card.

**Table 2:** Columbus Quantitative Data Collection

Survey type and timepoint	Dates	*N*	Response rate (total # sent survey)
SCHOLAR Pre-go-live (HPTs)	3/16/22–4/15/22	13	22.8%
SCHOLAR Post-go-live (HPTs)	6/1/22–7/15/22	10	17.5%
EMPIRIC Pre-Go-Live (Clinicians/Staff)	3/16/22–4/15/22	408	23%
EMPIRIC Post-go-live (Clinicians/Staff)	07/18/22–08/5/22	458	25.9%

In support of data merging, we drew HPT-relevant survey data from our team’s affiliated and parallel evaluation EMPIRIC, distributed to ~1770 Columbus employees. A total of 481 employees (27.2%) responded to the pre-go-live survey; 458 (25.9%) responded to the post-go-live survey (Table 2). The surveys gauged transition experiences and asked targeted questions about the EHR transition’s effect on VA’s training mission.

#### Key Measures

##### HPTs (SCHOLAR)

HPTs were asked about their experiences preparing to use Cerner (5 items), their perceptions of the current EHR (CPRS/VistA at pre-go-live, Cerner at post-go-live) adapted from the System Usability Scale (SUS)^19^ (18 items), satisfaction with their VA training experience (8 items), impact of the transition on clinical training (11 items), their ability to work with preceptors and patients, and overall experiences with the VA clinical learning environment adapted from the VA Learners Preceptor Survey.^20,21^ Survey items used a 5-point Likert response scale (response labels depended on question wording). HPTs were also asked clinical and demographic characteristics (see Table 3 for clinical characteristics, Table 4 in the Appendix for demographics). Five open-ended questions asked HPTs about training experience and general transition reflections.

**Table 3:** Characteristics of HPT Survey Respondents

Variable	Pre-go-live	Post-go-live
	*n*=13	*n*=10
VA clinical training program		
Medical student/resident	23%	30%
Nursing program	0%	0%
Optometry	0%	20%
Pharmacy	8%	10%
Psychology	0%	20%
Other	46%	10%
Missing	23%	10%
Hours allocated to VA clinical care per week		
< 10	23%	10%
10–19	8%	20%
20–29	23%	10%
30–39	0%	20%
40–49	15%	30%
50–60	8%	0%
60+	0%	0%
Missing	23%	10%
Time with VA		
< 1 month	0%	10%
1–3 months	23%	10%
4–6 months	8%	10%
7–12 months	46%	30%
12+ months	0%	30%
Missing	23%	10%
PGY		
Medical student	0%	30%
PGY-1	15%	10%
PGY-2	15%	10%
PGY-3	0%	20%
Post PGY-3	23%	20%
Missing	46%	10%

**Table 4:** Demographics of HPTs who Participated in the Pre-go-live and Post-go-live HPT Survey

Variable	Pre-go-live	Post-go-live
	*n*=13	*n*=13
Age (years)		
29 or younger	31%	69%
30–39	38%	23%
40–49	8%	0%
50–59	0%	8%
60+	0%	0%
Missing	23%	0%
Gender		
Female	23%	62%
Male	38%	38%
Prefer not to answer	15%	0%
Missing	23%	0%
Hispanic ethnicity		
No	62%	92%
Yes	8%	8%
Prefer not to answer	8%	0%
Missing	23%	0%
Race		
Asian	8%	31%
Black/African American	0%	8%
White	38%	54%
American Indian/Alaska Native	8%	0%
Asian	8%	8%
Some other race	8%	0%
Missing	31%	0%

##### Clinicians (EMPIRIC)

We report findings from two SUS-modified items assessing clinicians’ perceptions of EHR usability (“I find the [CPRS/VistA or Cerner] EHR very cumbersome to use” and “I feel very confident using the [CPRS/VistA or Cerner] EHR” with 5-point Likert response options). HPT supervisors were also asked, “How has the Cerner EHR implementation affected the VA’s training mission at your facility?”

#### Data Analysis

We conducted descriptive analysis of survey data by conducting top two box scoring, reflecting the proportion of HPTs who reported one of the two most favorable responses (e.g., agree or strongly agree). Quantitative survey data was analyzed in SAS statistical software.^22^

## Results

Trainees largely reported feeling the EHR transition holistically impacted every aspect of their HPT experience, comprehensively touching most learning spaces and processes, resulting in a challenging and disruptive experience. Three themes across accounts are notable: (1) challenges using the new EHR (including access issues, onboarding, and EHR training); (2) barriers to clinical learning and clinical skill acquisition tracking; and (3) impact on trainee interest in future VA careers. Each theme is presented with illustrative quotes and descriptive statistics.

### Challenges Accessing and Using the New EHR

#### EHR Access and Onboarding

The EHR transition disrupted the process of granting HPTs EHR access, complicating onboarding. Trainees could not use the new EHR until they had completed required trainings, creating serious delays when there were no post-start-date training timeslots available. Moreover, the time demands of repeated training across formats caused frustrations.

“It seems absurd that <HPTs> have <computer trainings> and then additional Cerner training when <we> do it more effectively <ourselves>… it’s a very poor use of trainees’ time” (*E222*_*Supervisor_1-month-pre-go-live).*

Supervisors often felt HPTs, employees, and Cerner staff were not “on the same page about onboarding” (*E210 Supervisor. 1 month pre-go-live*); even when provisions were granted, trainees frequently lacked access to tools like the practice EHR portal. HPTs thus had narrower windows for providing care.

“The <practice environment> was down last week… but <HPTs> have to complete all this training before they’re allowed in it. <For> the ones who just rotate through for a month… they’re not going to be able to really do anything” (*E210_Supervisor_1-month-pre-go-live*).

**Figure 2:**
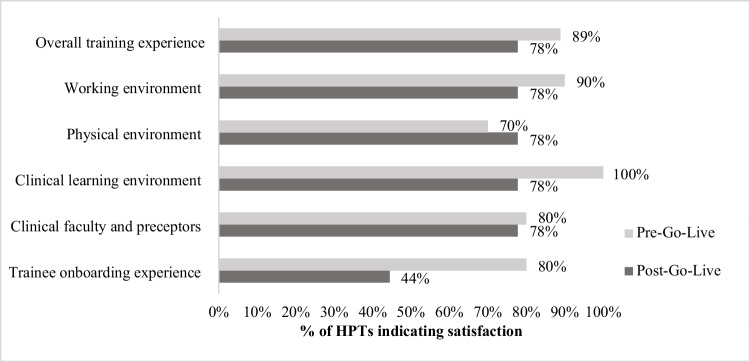
HPT satisfaction with VA learning environment at pre-go-live and post-go-live. The bar graph displays the proportion of HPTs who reported being “satisfied” or “very satisfied” for each survey item. The light grey bars represent the % of HPTs who were “satisfied” or “very satisfied” with the individual survey item at pre-go-live. The dark grey bars represent the % of HPTs who were “satisfied” or “very satisfied” with the individual survey item at post-go-live.

Survey data supported such sentiments: 80% of HPTs were satisfied with VA trainee onboarding experience pre-go-live, compared to 44% post-go-live (Fig. 2). Such barriers were particularly acute for shorter programs unable to accommodate delays. Some clinicians noted many trainees lacked access until 3 weeks into their four-week rotations; some longer-term trainees took over five weeks to get access.

#### EHR Training and Skill Acquisition

Delays, while themselves concerning, also exacerbated other findings, demonstrating the cumulative nature of EHR transition challenges. Like employees, HPTs were often dissatisfied with the new EHR training. Concerns included instructor quality, format, and ill-fit content for HPT needs.

Staff believed the combined volume of virtual and in-person trainings was a poor use of trainee’s time.

“<HPTs> described the online modules as ‘frustrating and redundant’ because it goes over the same information from in-person trainings" (*207_HPT_1-month-pre-go-live*)

Some supervisors used in-person sessions to supplement online training gaps; this was valued, though it did not remedy the lengthy time commitments.

“<HPTs are> required to do <online classes…> However we did set up training dates for <our> residents to come in <and> we're just going to walk them through the most common processes and things that they're going to do in the clinic” (*S203_Site Leader_1-month pre-go-live*).

**Figure 3:**
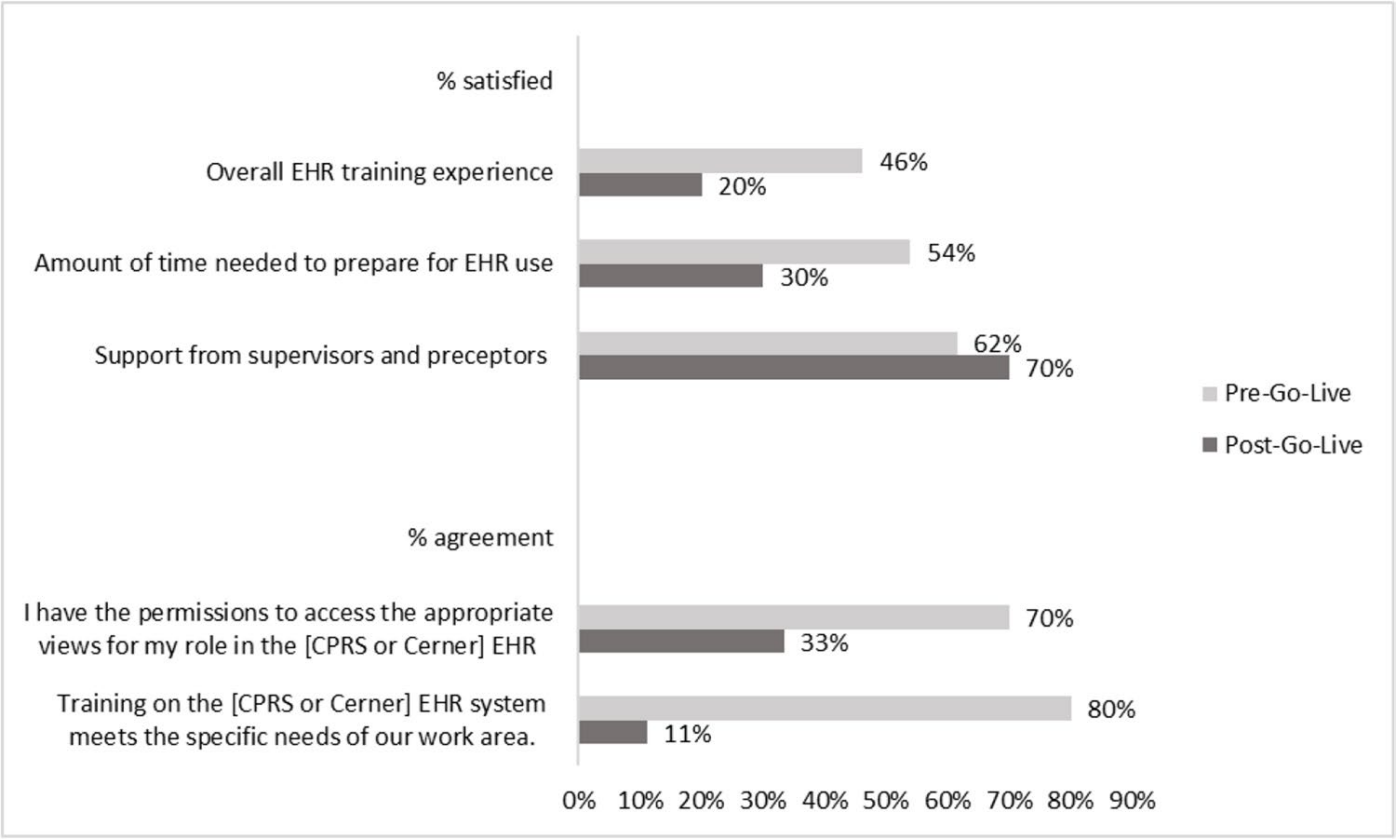
HPT perceptions of EHR training and usability at pre-go-live and post-go-live. The bar graph displays the proportion of HPTs who reported one of the two most favorable responses (i.e., satisfied or very satisfied; agree or strongly agree) for each survey item. The light grey bars represent the % of HPTs who were satisfied or agreed with the individual survey item at pre-go-live. The dark grey bars represent the % of HPTs who were satisfied or agreed with the individual survey item at post-go-live.

Trainees indicated training did not prepare them for role-specific workflows or tasks. Pre-go-live, 80% of HPTs reported that training met the specific needs of their work area; post-go-live, this fell to 11% (Fig. 3).

“The training that we received was really not sufficient to what we needed to do. It was more generalist training, < not> unique to our specific role” (*S202_HPT_2-months-post-go-live*).

HPT learning was limited when Cerner staff did not know the EHR or VA workflows, and subsequently could not instruct trainees on specific tasks. HPT and staff alike offered one suggestion:

“I honestly think that the people using the system<…> are the best teachers of how to use any system” (*S203_Site Leader_1-month-pre-go-live*).

Surveys indicated overall satisfaction with EHR training fell from pre- to post-go-live (Fig. 2). Despite this, a site leader shared that HPTs found trainings led by EHR-trained VA clinicians helpful, with some trainees sharing they “don’t know what <they> would have done” if the VA clinicians had not been present (2-months post-go-live), identifying improvement direction.

### Barriers to Clinical Learning and Tracking of Clinical Skill Acquisition

HPTs who completed training and had EHR access described three issues impacting their learning in fulfillment of VA’s training mission: (1) delayed EHR access limited HPT contributions to care, (2) EHR functionality issues limited reliability of HPT contributions to care, and (3) attendees own learning curves affected how well they could supervise incoming HPTs.

#### Delayed EHR Access Resulted in Less HPT Contribution to Clinical Care

Trainees and their supervisors noted that delayed EHR access limited their ability to be involved in patient care.

“We're not able to rely on a trainee to see patients for the first week. <Whereas> CPRS, <…> even if <something> wasn't set up right, by 48 hours, 95% of trainees are in the computer and ready to go.” (*S201_Site Leader_2-months-post-go-live*)

“On a <typical clinic> day we’d see 40-45 patients <…> going forward we see ten patients a day” (*S204_HPT_1-month-pre-go-live).*

Often, HPTs were required to complete extensive training on their own time to see patients in a timely manner:

“Residents were asked to complete our training outside of clinic whereas most providers were given their training time during clinic, so there’d be days where I would be completing Cerner training <until 11PM>” *(S208_HPT_1-month-pre-go-live).*

Surveys suggested such access delays resulted in a decline in satisfaction with the amount of time needed to prepare for EHR use from pre-go-live (54%) to post-go-live (30%) (Fig. 3).

When delays happened, HPTs had to observe instead of participating in the same level of clinical support that they did pre-transition. Collective delays led some programs to switch to “observation only.” In such cases, the risk becomes “cumbersome” for academic programs (*S201_Site Leader_1-month-pre-go-live*). They may have residents shadow instead of provide care, compromising training value. The general perception among HPTs and staff was that delays significantly complicated rotations, and kept HPTs from getting the learning experiences they anticipated.

#### Delayed EHR Functionality Result in Less HPT Contribution to Clinical Care

Participants described EHR functionality gaps that resulted in less HPT contribution to clinical care. Initially, procedures or services coded by HPTs did not contribute to their supervisors’ workload credits (VA measures identifying professionals’ care contributions) like pre-transition, resulting in supervisors failing to ‘get credit’ for supervisory work. As a workaround, supervisors were instructed to do coding (i.e., enter the billing codes associated with each service) themselves, increasing their work burden and limiting HPTs’ exposure to an important part of clinical care.

“<When HPTs code>, procedure workload credit doesn't seem to go to the attending doctor… instead of the resident coding, the attending does all the coding” (*S201_Site Leader_2-months-post-go-live*).

Additional post-access permission issues emerged. Interviewees described being unable to access images from other clinical areas, propose medications for supervisory review, or even meet basic skill development aims.

“As a senior resident the expectation is we see 10-15 people a day <…> we’re seeing 2-4 and a lot of it will be transcribing <…> it significantly is a decrease <…and> impact on the senior resident about to go into practice. I have a couple co-residents who are going into private practice, and this is the time they would hope to be operating a lot and fine-tuning their skills” (*S204_HPT_1-month-pre-go-live).*

Corresponding survey data demonstrated a drop in HPTs who reported having “permission to access appropriate views for their role in the EHR” from 70% pre-go-live to 33% post-go-live (Fig. 3).

#### Supervisors’ EHR Transition Struggles Impaired their Ability to Supervise HPTs

Even as trainees experience transition difficulties, findings from EMPIRIC (focusing on employee experience) suggested that HPT supervisors faced their own learning curves as they adapt to a new EHR. Such learning curves, compounded with the increased time it takes to conduct everyday tasks and general additions to workload from new workflows negatively impacts desire to supervise HPTs.

“Cerner in general takes much longer to document than CPRS so I am also spending less time teaching/educating due to EHR time” *(EMPIRIC_R44_Supervisor_2-months-post-go-live*).

High workloads, combined with decreased user confidence, lead some supervisors to consider it irresponsible to take on HPT supervision considering their own EHR struggles.

“I am reluctant to take on the additional work of having a student when I am still determining how to best use the system in my daily workflow especially when with patients” (E*MPIRIC_R143_Supervisor_2-months-post-go-live*).

There was a drop in confidence using the EHR from 82% at pre-go-live to 18% at post-go-live alongside an increase in users indicating that the EHR was cumbersome to use, from 14% pre-go-live to 84% post-go-live.

#### Barriers to Tracking Clinical Skill Acquisition

To successfully complete their training, HPTs must demonstrate acquisition of certain clinical skills, competencies, and time spent on patient care: metrics commonly reflected through EHR data (e.g., number and complexity of procedures performed, hours of patient care completed). With the EHR transition, VA HPT supervisors repeatedly noted barriers to collecting such training requirement data in the EHR. These challenges can directly impact HPT program completion and a program’s ability to meet accreditation standards. The ability to access correct HPT data from the EHR is also an OAA statutory reporting requirement to Congress.

“[We’re] missing clinical experience data. It looks like they're not doing procedures when they are” (*S201_Site Leader_2-months-post-go-live*).

### Decreased HPT Inclination to Pursue Future Careers at VA

Our final theme illustrates a cumulative effect of EHR-related disruptions on the HPT experience: collectively, the EHR transition made VA less appealing career-wise to some HPTs. A preliminary finding from the SCHOLAR survey, the proportion of HPTs expressing a likelihood to select VA for a future career decreased from 50% pre-go-live to 30% post-go-live. Qualitative data elaborated on potential contributory factors: HPTs often noted that the challenging EHR transition colored their perception of working at VA.

“It’s definitely impacted morale. I know for myself and other interns we’re ready to get out of here… with Cerner it’s like a chore to stay here” (S*202_HPT_2-months-post-go-live*).

“Overall, I have enjoyed working in the VA. However, I believe Cerner has negatively impacted my training and I am excited to be leaving the VA” (S*CHOLAR_R4_HPT_2-months-post-go-live*).

Despite these impacts, HPT experiences and overall satisfaction with VA remained remarkably high. HPTs survey respondents largely remained satisfied with VA training (78% post-go-live), compared to 89% pre-go-live. Moreover, HPTs reported higher satisfaction with EHR training support from supervisors and preceptors post-go-live (70%) than pre-go-live (62%) (Fig. 3) and maintained high levels of general satisfaction with clinical faculty and preceptors from pre- (80%) to post-go-live (78%; Fig. 2). Despite EHR transition challenges, many HPTs found value in their VA training.

## Discussion

Our evaluation addresses a gap in EHR transition literature by identifying widespread HPT challenges that impacted the quality of HPTs’ training experience due to lack of consideration of their unique needs. HPTs face challenges not experienced by employees such as completing training requirements across short multiple rotations and credit tracking complications. Such findings can enhance the emerging body of literature on HPT EHR transitions and present suggestions for health care systems training HPTs planning for and undergoing an EHR transition.

Initial EHR transition shapes subsequent EHR adoption and confidence. Inadequate training can result in inappropriate EHR use and adverse events^23,24^, including medication error and other patient safety concerns.^25,26^ Training offers informative experiences that can shape where trainees choose to practice^27^ and training designed for employees may not fit HPTs’ distinct needs.

Following training, HPTs reported delayed EHR access that limited their ability to provide patient care, as well as a lack of reliable data needed to fulfill their training requirements. EHR transitions present a unique challenge for health systems providing HPT training because attendees themselves potentially lack expertise in the new EHR and are thus learning themselves. Combined, these factors can profoundly impact attendees’ ability to reliably support, supervise and educate HPTs. Site leadership expressed concerns that in the face of such problems, HPT programs—even outside VA—could switch to observation only or potentially discontinue entirely.

These negative experiences can lead to burnout, low morale, and decreased likelihood of selecting a system for future work.^28^ It is not surprising that HPTs interested in pursuing VA careers declined compared to institutional pre-transition data, which showed trainees were 2–5 times more likely to consider working for VA after rotations.^29^ Health systems planning for EHR transitions should engage clinicians in developing training materials that meet HPTs needs and training requirements^30^ and, ideally, have clinicians familiar with the system’s workflows and responsibilities conduct training rather than vendor staff. Adequate support from system leadership to allow individuals to accommodate training responsibilities into their already full workdays is critical.^31^

Widespread negative impacts can impair the VA’s ability to uphold its mandates to provide timely patient care and to train HPTs for both its and the nation’s needs.^12^ Early, ongoing, and robust coordination among key health system stakeholders (e.g., local/system-wide educational and clinical leadership, academic affiliates, EHR vendors/developers) is critical in ensuring HPT’s educational needs, including timely onboarding and provisioning. Consequently, OAA stated a goal of HPT EHR access from day one of rotations and developed recommendations to improve VA HPT EHR integration based on these findings (Table 5 in the Appendix). Integration of these recommendations can ensure VA’s educational training capacity, support HPT ability to provide care, and maintain recruitment and retention of personnel.

**Table 5:** OAA Recommendations for VA Sites Undergoing EHR Transitions

OAA Recommendations for VA	
• HPT EHR access should be simultaneous with training and submitted on a rolling basis	
• HPTs should not be required to complete instructor-led training	
• The transition to computer-based training for all HPT roles should be completed, and all HPT training should be shortened and consolidated into a single course for each role	
• The EHR training curriculum for HPTs should be formatted to allow hosting on the VA Train website outside the VA firewall. The legacy system CPRS/VistA training is currently hosted on that site	
• Technological avenues available should be used to support asynchronous learning.	
• The local VA Educational Office and Designated Education Officer (DEO) should be included in all deployment activities and Change Leadership Teams to ensure HPT issues are addressed	
• When training curriculums are developed or modified, time constraints on HPTs should be considered, and ease of use should be prioritized	
• These improvement recommendations and processes can inform both VA and non-VA systems’ transitions through the mechanism of robust at-the-elbow training	
• Local coordinator positions should be created to to help mitigate training assignment and provisioning challenges; tracking individuals during the EHR provisioning process; and providing adequate support during go-live periods (especially for short-term rotations).	

Other systems can similarly learn from these considerations. First, educational leadership should participate in the entire lifecycle of deployment planning alongside health systems, particularly in coordinating training duration and timing. Second, HPT EHR training should leverage technology to allow for asynchronous learning and minimize ill-fit/repetitive content. Additionally, creating local coordinator positions can mitigate training assignment and provisioning challenges. Finally, comprehensive tracking through provisioning can promptly identify in-need individuals.

Data come from voluntary VA HPT and clinician participants from one outpatient VA Medical Clinic, opening the possibility of self-selection bias. Trainee experiences at other VA sites, inpatient settings, and non-VA sites may differ. Additionally, we engaged employees and HPTs across a high-stress event; snowballing recruitment reach was limited by individuals declining to participate due to high workloads and limited time to engagement. In such cases, many individuals shared a desire to participate while acknowledging the need to prioritize their regular work duties during transition.

Our small sample of interviewees (*n*=8) may not encompass a diversity of perspectives from HPTs and supervisors across clinical areas and training programs; we accordingly exercised caution in interpreting findings for contexts and perspectives yet to be examined. Pursuing saturation was not feasible given study timeframe, available resources, and potential participant pool time commitments. The low sample sizes for HPT engagement are a limitation of our findings; however, merging data streams across quantitative surveys, interviews, and survey open-ended questions strengthened the reliability of findings. Because we did not reach thematic saturation in the interviews we conducted, future interviews with HPTs experiencing EHR transitions are likely to illuminate additional themes and insights while forming a more robust sample size.

## Conclusions

Although the VA is the largest provider of health professional training in the USA, it is only one of many systems that provide training and rely on HPTs to provide patient care. Identifying, understanding, and proactively addressing the unique challenges that EHR transitions pose for HPTs is critical to ensuring effective health care training. Health systems implementing new EHRs should anticipate structural and functional barriers that can prevent HPTs from providing care and obtaining required training metrics. Such measures can mitigate HPT job dissatisfaction and compromises to quality patient care during EHR transitions. Further research is needed to assess efforts to improve HPT EHR transitions, understand how provider transition experiences further complicate HPT experiences, and develop strategies that not only mitigate negative impacts, but also enhance health professionals’ training.

### Supplementary Information

ESM 1 (DOCX 31 kb)
